# Signatures of seaway closures and founder dispersal in the phylogeny of a circumglobally distributed seahorse lineage

**DOI:** 10.1186/1471-2148-7-138

**Published:** 2007-08-15

**Authors:** Peter R Teske, Healy Hamilton, Conrad A Matthee, Nigel P Barker

**Affiliations:** 1 Molecular Ecology and Systematics Group, Botany Department, Rhodes University, 6140 Grahamstown, South Africa; 2 Evolutionary Genomics Group, Department of Botany and Zoology, Stellenbosch University, Private Bag X1, Matieland 7602, South Africa; 3 Research Division, California Academy of Sciences, 875 Howard St., San Francisco, CA 94103, USA

## Abstract

**Background:**

The importance of vicariance events on the establishment of phylogeographic patterns in the marine environment is well documented, and generally accepted as an important cause of cladogenesis. Founder dispersal (i.e. long-distance dispersal followed by founder effect speciation) is also frequently invoked as a cause of genetic divergence among lineages, but its role has long been challenged by vicariance biogeographers. Founder dispersal is likely to be common in species that colonize remote habitats by means of rafting (e.g. seahorses), as long-distance dispersal events are likely to be rare and subsequent additional recruitment from the source habitat is unlikely. In the present study, the relative importance of vicariance and founder dispersal as causes of cladogenesis in a circumglobally distributed seahorse lineage was investigated using molecular dating. A phylogeny was reconstructed using sequence data from mitochondrial and nuclear markers, and the well-documented closure of the Central American seaway was used as a primary calibration point to test whether other bifurcations in the phylogeny could also have been the result of vicariance events. The feasibility of three other vicariance events was explored: a) the closure of the Indonesian Seaway, resulting in sister lineages associated with the Indian Ocean and West Pacific, respectively; b) the closure of the Tethyan Seaway, resulting in sister lineages associated with the Indo-Pacific and Atlantic Ocean, respectively, and c) continental break-up during the Mesozoic followed by spreading of the Atlantic Ocean, resulting in pairs of lineages with amphi-Atlantic distribution patterns.

**Results:**

Comparisons of pairwise genetic distances among the seahorse species hypothesized to have diverged as a result of the closure of the Central American Seaway with those of published teleost sequences having the same distribution patterns show that the seahorses were among the last to diverge. This suggests that their cladogenesis was associated with the final closure of this seaway. Although two other divergence events in the phylogeny could potentially have arisen as a result of the closures of the Indonesian and Tethyan seaways, respectively, the timing of the majority of bifurcations in the phylogeny differed significantly from the dates of vicariance events suggested in the literature. Moreover, several divergence events that resulted in the same distribution patterns of lineages at different positions in the phylogeny did not occur contemporaneously. For that reason, they cannot be the result of the same vicariance events, a result that is independent of molecular dating.

**Conclusion:**

Interpretations of the cladogenetic events in the seahorse phylogeny based purely on vicariance biogeographic hypotheses are problematic. We conclude that the evolution of the circumglobally distributed seahorse lineage was strongly influenced by founder dispersal, and suggest that this mode of speciation may be particularly important in marine organisms that lack a pelagic dispersal phase and instead disperse by means of rafting.

## Background

Bifurcations in a phylogeny can be explained by two modes of allopatric speciation: vicariance [1] (the establishment of a dispersal barrier separating regional units of a previously continuously distributed species) and founder dispersal [2-4] (long-distance dispersal of a small number of individuals from a source population followed by founder effect speciation in the absence of additional gene flow). Although most biogeographers consider both vicariance and founder dispersal to be important causes of cladogenesis, speciation as a result of dispersal is sometimes rejected or considered irrelevant noise on the basis of its sporadic nature. It is argued that in the majority of cases in which dispersal has been invoked, it is considered to have affected only individual species rather than the entire fauna of a particular region [5].

Cladogenesis as a result of founder dispersal may be more important in seahorses (genus *Hippocampus*) than in many other marine organisms studied to date, because of their life-histories and means of dispersal [6]. Seahorses use a prehensile tail to hold on to objects that may serve as rafts, such as floating seaweed [7,8]. As macrobenthic prey tends to be abundant on these rafts [9], displaced seahorses are likely to survive for a considerable amount of time. In most species, pregnant male seahorses can have brood sizes of up to 100–300 individuals [10], suggesting that a sufficiently large number of closely related individuals may arrive simultaneously at a new habitat to establish themselves. As additional recruitment from the source population is unlikely (because long-distance dispersal along the same route is likely to be rare), lack of gene flow coupled with genetic drift may eventually result in speciation. The combination of seahorses rarely dispersing through the open ocean, but surviving well in it and having a high potential of successfully founding new populations, makes them interesting models for studying the relative importance of vicariance and founder dispersal in marine organisms that disperse by means of rafting.

### Molecular dating of marine organisms' phylogenies

Recent advances in the field of model-based analyses have considerably improved the level of confidence in time estimates obtained from molecular data [11]. Variations in evolutionary rates can be accounted for, and uncertainties with regard to calibration points can be incorporated by specifying upper and/or lower limits for a particular divergence event. Nonetheless, in recent reviews of molecular biogeography and molecular dating, Heads [12,13] criticized studies whose results supported cladogenesis as a result of founder dispersal on the basis of questionable molecular dating. Molecular dating can be performed using three methods of calibration: a) dating of the root of the phylogeny of a particular taxon by using the age of the taxon's oldest known fossil; b) dating of the age of a taxon present on a volcanic island by using the age of the island and c) dating of the cladogenic event that gave rise to two lineages present on either side of a geological barrier by using the time when the barrier formed. Heads [13] rejected the first two methods for the following reasons. Firstly, new fossils are often found that are considerably older than the previously known oldest fossil of a particular taxon, and it is thus impossible to be certain whether the oldest fossil of a particular taxon has indeed been found. The method can be considered particularly problematic in the case of shallow water marine organisms, whose fossil record is often fragmented [14,15]. Seahorses present a case in point, because their fossils are known from only two sites in the northern Mediterranean [16,17]. Secondly, dating by means of the age of volcanic islands can be problematic because such islands are located on subduction zones where new islands are created and old ones disappear continuously, suggesting that an extant species occurring on a volcanic island may be older than its habitat.

### Molecular dating using vicariance events

Two major types of vicariance events are potentially useful to calibrate molecular clocks of marine organisms: seaway closures and continental break-up. The best documented vicariance event that impacted on the biogeography of marine species is the closure of the Central American Seaway. The rising of the Isthmus of Panama during the Pliocene isolated the tropical western Atlantic and eastern Pacific oceans [18-20] and resulted in the divergence of formerly continuously distributed species, many of which have remained morphologically similar and are thus readily recognizable as sister taxa. For that reason, the majority of studies on marine species have used the closure of the Central American Seaway as a calibration point [21-23]. However, the utility of such geminate species for calibrating molecular clocks can be nonetheless be problematic. Firstly, some of the species that have been identified as sister taxa may have diverged prior to the final closure of the Central American Seaway (3.1 – 3.5 mya [19]), which results in overestimates of mutation rates [22-24]. Such earlier divergence may have been the result of the oceanographic changes associated with the rising of the isthmus before final seaway closure, or originations resulting from changes in carbonate levels as a result of seaway constriction [25,26]. Secondly, incomplete taxon sampling may result in the wrong species being identified as sister taxa [27].

Few studies on marine organisms have sampled lineages with circumglobal distributions [28,29]. For these, additional seaway closures could be used as calibration points, which may result in greater precision of molecular dating. The closure of the Tethyan Seaway, which once connected the Atlantic Ocean with the Indo-Pacific via the Mediterranean, has been used comparatively rarely to date phylogenies [30,31]. The reason for this may be that the occurrence of both tectonism and climate change (resulting in cyclic fluctuations in the sea level) makes dating of the vicariance event that severed the link between the Mediterranean and the Indo-Pacific problematic, and the exact date of this seaway's final closure is disputed. Drooger [32] considered it to have taken place during the Late Oligocene (23.8 – 28.5 mya), Adams et al. [33,34] suggested a Late Early Miocene closure (18.4 – 20.5 mya), and Rögl and Steininger [35,36] argued for a temporary re-opening during the Middle Miocene (14.8 – 18.4 mya) followed by complete closure 11.2 – 14.8 mya. Adams et al. [37] rejected the evidence for both the Late Oligocene and Middle Miocene closures on the basis of questionable dating. The Middle Miocene date for a temporary re-opening of the seaway [35,36] is nevertheless widely accepted, although some marine organisms from the Indian Ocean do not seem to have dispersed through the Tethyan seaway during this time [38].

Recent genetic work identified the existence of marine sibling species associated with the Indian and Pacific Oceans, respectively, whose distributions sometimes overlap in Indonesia [6,39-43]. The divergence between some of these has been attributed to temporary closure events of the Indonesian Throughflow, which presently connects the Indian and Pacific oceans. Complete and long-lasting closure events were estimated to have taken place during the Middle Miocene (15 – 17 mya [44]) and during the Late Miocene (7.5 – 9.9 mya [45] or 7.0 – 9.5 mya [46]). A further closure of the Indonesian Seaway took place during the Pliocene (3 – 4 mya [47]). Lastly, tectonic uplifts and lowered sea level during Pleistocene glaciations resulted in restricted exchange between Indian Ocean and West Pacific Ocean faunas [48,49].

In addition to distribution patterns that may have arisen as a result of the three seaway closure events, the presence of sister species on either side of the Atlantic Ocean could be interpreted as being the result of continental break-up. Rosen [50] attributed such patterns to sea-floor spreading and widening of the Atlantic Ocean, following the separation of Africa and South America no later than approximately 84 mya [51].

### The relative importance of vicariance and founder dispersal

**Figure 1:**
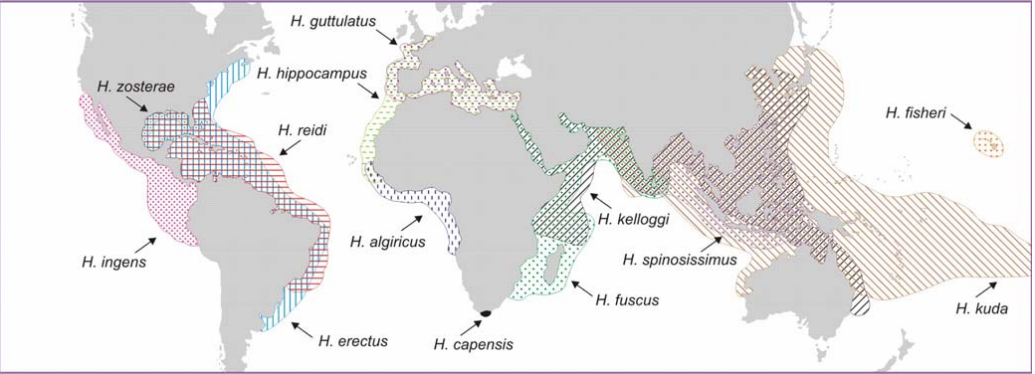
**Seahorse distribution ranges**. Geographic distributions of species associated with the circumglobally distributed seahorse lineage (after Lourie et al. [61]).

In the present paper, we investigated the relative importance of vicariance and founder dispersal as modes of allopatric speciation that may have impacted on the phylogeny of a circumglobally distributed seahorse lineage (Fig. 1), while accounting for the abovementioned uncertainties regarding molecular dating of the phylogenies of marine organisms. This particular lineage was chosen because its wide distribution may either indicate ancient vicariance or large-scale dispersal, and because it may have been affected by more than one vicariance event. Exact phylogenetic relationships are not fully understood, but all species associated with this lineage have been identified using molecular methods [52,53], ensuring complete taxon sampling. Other seahorse lineages are less useful for this purpose, because they have more restricted distributions, phylogenetic relationships are comparatively poorly resolved, and a recent increase in species descriptions makes it likely that not all species have yet been identified [52-54].

Without knowledge of divergence times, the distribution patterns of several pairs of lineages within the circumglobal clade could be interpreted as being the result of vicariance. These include Eastern Pacific vs. Atlantic species (whose divergence could be the result of the closure of the Central American Seaway), Indian Ocean vs. West Pacific species (Indonesian Seaway closure), Atlantic vs. Indo-Pacific species (Tethyan Seaway closure) and amphi-Atlantic species pairs (spreading of the Atlantic Ocean).

Vicariance biogeographers have objected to founder dispersal hypotheses because the sporadic nature of such events does not make them falsifiable [55]. Although the establishment of ocean currents has in some cases resulted in repeated directional long-distance dispersal [56,57], whether or not a species becomes established in a new habitat depends considerably on its dispersal abilities and life history characteristics. Even if a large number of species share a pattern resulting from founder dispersal, it is unlikely that they all would have colonized a particular habitat contemporaneously, and molecular dating using founder dispersal is thus less precise than molecular dating using vicariance events. However, if vicariance hypotheses can be rejected, then founder dispersal should be supported by default [5]. In this study, we estimated the ages of divergence events of seahorse lineages with geminate distribution patterns by using the well-documented closure of the Central American Seaway as a primary calibration point. Vicariance biogeographic interpretations of these are challenged if a) divergence time estimates are significantly different from the dates of vicariance events suggested in the literature and b) divergence events in different positions in the phylogeny that have resulted in the same distribution patterns of sister lineages have occurred at different times.

## Results

### Phylogenetic reconstructions

**Figure 2:**
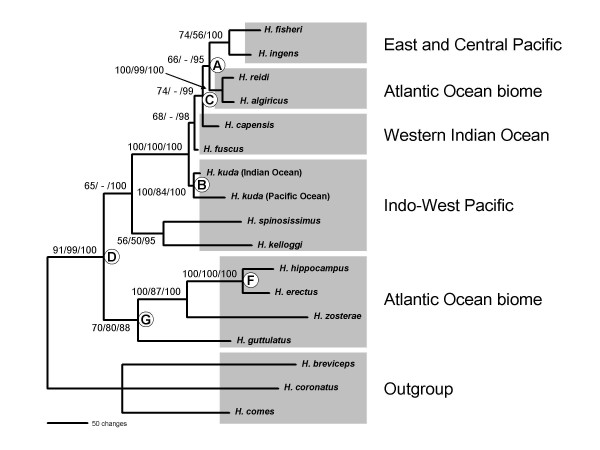
**Phylogeny of the circumglobal seahorse lineage**. The phylogenetic tree with the highest likelihood score reconstructed by means of likelihood ratcheting. The data matrix comprised five partitions: mitochondrial control region, cytochrome *b *and 16S rRNA, and nuclear S7 intron and Aldolase. Associations of lineages with biogeographic regions are indicated. Nodal support is indicated by three numbers; these represent bootstrap values from maximum likelihood searches, jackknife support from parsimony searches, and posterior probabilities from Bayesian Inference. Hyphens indicate clades that were not recovered using parsimony. White circles indicate divergence events that may have resulted from vicariance events. Letters within these correspond to those in Table 2.

A maximum likelihood phylogenetic tree reconstructed from sequence data of three mitochondrial and two nuclear markers recovered six major clades that were each associated with a distinct biogeographic region (Fig. 2). A congruent tree was recovered with Bayesian Inference, and most nodes were supported by significant posterior probabilities. The most parsimonious tree, on the other hand, did not recover some of the lineages, but none of the nodes that differed from the other two phylogenies had high support (all jackknife values <56). When a parsimony tree was constructed using the two most rapidly mutating markers only (control region and cytochrome *b*), then node A was also recovered.

The distribution patterns of several lineages in the phylogeny indicate that they could be the result of seaway closures or spreading of the Atlantic Ocean (indicated by letters A – G). In several cases, the same distribution patterns were recovered in different positions in the tree. Firstly, a split into an Atlantic and an Indo-West Pacific lineage was recovered twice. The first event (node D) resulted in a split into an Atlantic Ocean group comprising *Hippocampus hippocampus*, *H. erectus*, *H. zosterae *and *H. guttulatus*, and a group whose basal split resulted in an Indo-West Pacific lineage comprising *H. spinosissimus *and *H. kelloggi *and a lineage comprising all remaining species (present in the Indo-West Pacific, Atlantic Ocean and East/Central Pacific). The fact that Indo-West Pacific species are present in both of the latter lineages suggests that this group may have originated in this region. The second event that resulted in an Atlantic Ocean and an Indo-West Pacific lineage is the split defined by node C, resulting in the divergence of *H. capensis *(western Indian Ocean) and the Atlantic/eastern Pacific group comprising *H. ingens*, *H. fisheri*, *H. reidi *and *H. algiricus*. Amphi-Atlantic distribution patterns were recovered three times, the most basal being a split between the eastern Atlantic *H. guttulatus *and a group including the western Atlantic species *H. erectus *and *H. zosterae *(node G; this group also includes the eastern Atlantic species *H. hippocampus *in a derived position), followed by a later amphi-Atlantic split between *H. erectus *and *H. hippocampus *(node F). The divergence between *H. reidi *and *H. algiricus *resulted in a further amphi-Atlantic distribution pattern (node E).

### Molecular dating

**Figure 3:**
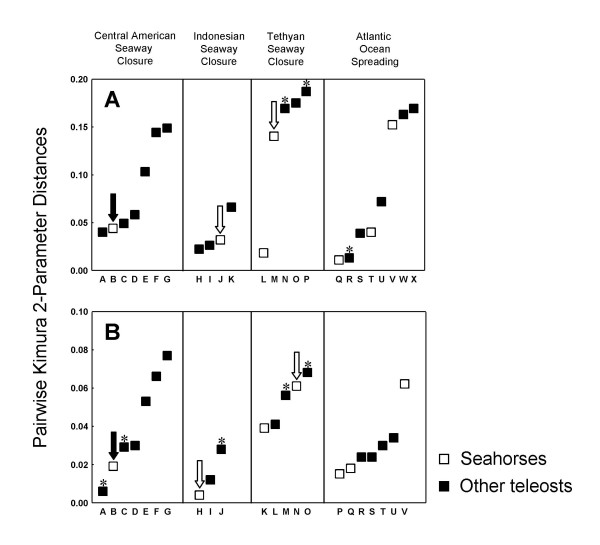
**Genetic distances among geminate teleost species**. Pairwise Kimura 2-Parameter distances [104] between lineages of seahorses (white squares) and other teleost species (black squares) whose present-day distribution patterns indicate that they may have diverged as a result of the closures of the Central American, Indonesian, or Tethyan seaways, or as result of continental break-up and spreading of the Atlantic Ocean. **A**: Cytochrome *b *sequences; **B**: 16S rRNA sequences. Letters represent teleost lineages listed in Table 1. The black arrow indicates the pairwise distance between the two seahorses lineages hypothesized to have diverged as a result of closure of the Central American Seaway. These lineages were used to estimate divergence times in Table 2. White arrows indicate distances between lineages whose divergence time estimates matched published dates for the closures of the Indonesian and Tethyan seaways (Table 2). Asterisks indicate teleost lineages whose evolutionary rate differed from that of the seahorses (**A**: N), whose rates were not tested because a different portion of 16S rRNA was sequenced (**B**: A, C, M, O) or which were used as outgroup in relative rate tests (**A**: P, R; **B**: J).

To explore the feasibility that the seahorse lineages present on either side of the Isthmus of Panama diverged as a result of Central American Seaway closure, we compared pairwise genetic distances between them with genetic distances between other geminate teleost lineages present on both sides of the Isthmus of Panama (Table 1, Fig. 3). We hypothesized that if the divergence of the seahorse lineages was linked to the final closure of the seaway, then the pairwise distance between them should be among the lowest of all the teleost lineages investigated. Pairwise distances were also calculated for pairs of teleost lineages that could have arisen as a result of the other three vicariance events relevant to the circumglobally distributed seahorse lineage by virtue of their distribution patterns. Using relative rate tests, it was found that only one of the teleost genera whose species have distribution patterns identical to those of the geminate seahorse lineages was characterized by a significantly different rate (*Lethrinus *vs. *Hippocampus *cytochrome *b*: difference in the rate of non-synonymous sites = -0.031 ± 0.015 [S.D.], p = 0.039). The rate of 16S rRNA of a number of genera could not be compared with the seahorses, because a different portion of this marker had been sequenced. Pairwise distances between these and the seahorses, as well as those of *Lethrinus *and *Albula *(used as outgroup) and the seahorses, are nonetheless shown in Fig. 3 and are indicated with asterisks.

**Table 1:** Cytochrome *b *and 16S rRNA sequences of various teleost lineages whose geographic distributions may have resulted from the closures of the Central American, Indonesian and Tethyan seaways, or from continental break-up and spreading of the Atlantic Ocean. Pairwise Kimura 2-Parameter distances between lineages were plotted in Fig. 3.

Molecular marker	Letter in Fig. 3	Lineage 1	Lineage 2	Accession numbers	Reference
Cytochrome *b*	A	*Selene peruviana*	*S. setapinnis*	AF363743/AF363745	118
	B	*Hippocampus ingens/fisheri*	*H. reidi/algiricus*	(see Table 3)	(this study)
	C	*Merluccius albidus*	*M. productus/gayi*	AY821666/AY821670/AY821771	Perez et al., unpubl.
	D	*Aulostomus chinensis*	*A. maculatus*	AF327455/AF327456AY786433	28
	E	*Centropomus viridis*	*C. undecimalis/poeyi*	AF018599–AF018629	23
	F	*Ophioblennius atlanticus*	*O. steindachneri*	AF323030-AF323038	72
	G	*Strongylura marina*	*S. exilis*	AF231641/AF231647/AF231653/AF231654	97
	H	*Chaetodon paucifasciatus*	*C. rhombochaetodon *complex	U23585–U23733	39
	I	*Chaetodon guttatissimus*	*C. multicinctus/punctatofasciatus*	U23585–U23733	39
	J	*Hippocampus kuda *(Indian Ocean)	*H. kuda *(Pacific Ocean)	(see Table 3)	(this study)
	K	*Pterois miles*	*P. volitans*	AJ429419–AJ429433	42
	L	*Hippocampus capensis*	*H. ingens/fisheri/reidi/algiricus*	(see Table 3)	(this study)
	M	*Hippocampus guttulatus/zosterae/erectus/hippocampus*	*H. spinosissimus/kelloggi/kuda/fuscus/capensis/reidi/algiricus/ingens/fisheri*	(see Table 3)	(this study)
	N	*Lethrinus atlanticus*	*Lethrinus *Indo-Pacific lineage	AF812251–AF812271	119
	O	*Thalassoma *(Atlantic Ocean lineage)	*Thalassoma *(Indo-Pacific lineage)/*Gomphosus*	AY328857–AY328885	120
	P	*Albula vulpes*/*glossodonta*/A/B/C/E	*A. neoguinaica*/D	AF311751–AF31171	121
	Q	*Hippocampus reidi*	*H. algiricus*	(see Table 3)	(this study)
	R	*Albula *sp. B (West Atlantic)	*Albula *sp. B. (East Atlantic)	AF311751–AF311756	121
	S	*Strongylura timucu*	*S. senegalensis*	AF231653/AF231654	97
	T	*Hippocampus erectus*	*H. hippocampus*	(see Table 3)	(this study)
	U	*Sparisoma axillare/rubripinne *(West Atlantic)	*S. rubripinne *(East Atlantic)	DQ457034–DQ457036	98
	V	*Hippocampus guttulatus*	*H. zosterae/erectus/hippocampus*	(see Table 3)	(this study)
	W	*Nicholsina usta usta*	*N. usta collettei*	DQ457022/DQ457023	98
	X	*Thalassoma norohanum/bifasciatum*	*T. newtoni/sancthelenae/ascensionis/pavo*	AY328861/AY328863/AY328876/AY328877/AY328882	120
					
16S rRNA	A	*Holocanthus passer*	*H. bermudensis*	AY530857/AY530867	61
	B	*Hippocampus ingens/fisheri*	*H. reidi/algiricus*	(see Table 3)	(this study)
	C	*Pomacantus paru/arcuatus*	*P. zonipectus*	AY530852/AY530868/AY530874	61
	D	*Centropomus ensiferus*	*C. robalito*	U85008/U85011	23
	E	*Centropomus viridis*	*C. undecimalis/poeyi*	U85012/U85013/U85014	23
	F	*Strongylura exilis*	*S. marina*	AF231515/AF231521	86
	G	*Aulostomus chinensis*	*A. maculatus*	AY141423/AY538973	122,123
	H	*Hippocampus kuda *(Indian Ocean)	*H. kuda *(Pacific Ocean)	(see Table 3)	(this study)
	I	*Pterois miles*	*P. volitans*	AJ429402–AJ429404/AJ429409–AJ429411	42
	J	*Albula vulpes*	*Albula glossodonta*	AY857934AP002973	Seyoum et al., unpubl.; 124
	K	*Hippocampus capensis*	*H. reidi/algiricus/ingens/fisheri*	(see Table 3)	(this study)
	L	*Thalassoma *(Atlantic Ocean lineage)	*Thalassoma *(Indo-Pacific lineage)/*Gomphosus*	AY328984–AY329012	120
	M	*Holacanthus passer/bermudensis/ciliaris/tricolor*	*Pygoplites diacanthus*	AY530847/AY530861/AY530864/AY530867/AY530873	61
	N	*Hippocampus guttulatus/zosterae/erectus/hippocampus*	*H. spinosissimus/kelloggi/kuda/fuscus/capensis/reidi/algiricus/ingens/fisheri*	(see Table 3)	(this study)
	O	*Pomacanthus semicirulatus/asfur/sexstriatus*	*P. paru/arcuatus/zonipectus*	AY530844/AY530852/AY530858/AY530868/AY530874	61
	P	*Hippocampus reidi*	*H. algiricus*	(see Table 3)	(this study)
	Q	*Hippocampus erectus*	*H. hippocampus*	(see Table 3)	(this study)
	R	*Sparisoma axillare/rubripinne *(West Atlantic)	*S. rubripinne *(East Atlantic)	(Not yet available)	98
	S	*Strongylura timucu*	*S. senegalensis*	AF231526/AF231527	97
	T	*Nicholsina usta usta*	*N. usta collettei*	Not on GenBank	98
	U	*Thalassoma norohanum/bifasciatum*	*T. newtoni/sancthelenae/ascensionis/pavo*	AY328988/AY328990,AY329003/AY329004/AY329009	120
	V	*Hippocampus guttulatus*	*H. zosterae/erectus/hippocampus*	(see Table 3)	(this study)

Comparisons of mean K2P distances between pairs of seahorse lineages that may have diverged as a result of vicariance events with those of other teleosts having congruent distribution patterns indicate that the seahorse lineages defined by node A in Fig. 2 (Central American Seaway closure) diverged comparatively recently. We considered this to be evidence for a Pliocene divergence of this lineage as a result of the final closure of the Central American Seaway, an event that is considered to have occurred no earlier than 4.6 mya. The mean genetic distance among cytochrome *b *sequences of the lineage defined by node B (Indonesian Seaway closure) was slightly lower than that of the lineage defined by node A, whereas the genetic distance among 16S rRNA sequences was distinctly lower. K2P distances among control region sequences (not shown) were also slightly lower for Indonesian Seaway divergence than for Central American Seaway divergence (0.036 and 0.043, respectively), indicating that this event may have taken place during the Late Pliocene or Early Pleistocene. Present-day distribution patterns of several pairs of lineages in the seahorse phylogeny could be interpreted as having resulted from the remaining two vicariance events (two in the case of Tethyan seaway closure and three in the case of continental break-up during the Mesozoic). Mean pairwise K2P distances between these differed considerably, as did distances between other teleost lineages having the same distribution patterns.

Divergence time estimates among seahorse lineages were obtained using a relaxed molecular clock method [58]. When the final date for the closure of the Central American seaway (3.1 – 3.5 mya) is accepted as the date when the transisthmian seahorse lineages diverged, based on the considerations in the previous paragraph, then only two divergence estimates of nodes defining species pairs whose distribution patterns indicate that they could have resulted from vicariance events, matched the dates suggested in the literature (Table 2). Firstly, the Indian Ocean and West Pacific lineages of *H. kuda *(node B) were estimated to have diverged 3.73 ± 0.29 mya, which indicates that this cladogenic event may have resulted from the closure of the Indonesian Seaway 3 – 4 mya [46]. Secondly, the species defined by node D were estimated to have diverged 15.12 ± 3.46 mya, which matches the temporary re-opening of the Tethyan seaway during the Middle Miocene (14.8–18.4 mya) followed by complete closure 11.2 – 14.8 mya [34,35]. However, confidence intervals for this estimate are comparatively large (9.85 – 23.26 mya), and the earlier date of 18.4 – 20.5 mya [36] must also be considered feasible.

**Table 2:** Divergence time estimates among seahorse lineages whose cladogenesis may have been the result of vicariance events by virtue of the present-day distribution patterns of their species.

	Node					
	
Calibration range	B	C	D	E	F	G
3.1 – 3.5	3.73 ± 0.29	3.67 ± 0.42	15.12 ± 3.46	1.47 ± 0.53	5.33 ± 1.79	14.60 ± 3.37
	(2.26 – 5.84)	(3.18 – 4.76)	(9.85 – 23.26)	(0.54 – 2.61)	(2.64 – 9.47)	(9.53 – 22.49)
3.1 – 4.6	4.26 ± 1.15	4.16 ± 0.66	16.60 ± 4.00	1.66 ± 0.63	6.01 ± 2.13	16.05 ± 3.89
	(2.46 – 6.94)	(3.24 – 5.70)	(10.50 – 26.05)	(0.60 ± 3.04)	(2.89 – 11.14)	(10.09 – 25.22)
3.1 – 8.5	5.15 ± 1.92	5.03 ± 1.50	19.07 ± 5.76	2.02 ± 0.94	7.18 ± 3.07	18.46 ± 5.63
	(2.60 – 9.98)	(3.27 – 8.75)	(11.04 – 33.05)	(0.66 – 4.31)	(3.07 – 14.87)	(10.60 – 32.16)

Divergence times were estimated using the program MULTIDIVTIME [102] under the assumption that the closure of the Central American Seaway (Node A in Fig. 2) resulted in the divergence of two sister lineages associated with the eastern/central Pacific (*Hippocampus ingens *and *H. fisheri*) and Atlantic Oceans (*H. reidi *and *H. algiricus*), respectively. Phylogeographic distribution patterns may have been the result of the following vicariance events. Node B (Indian Ocean vs. West Pacific): Closure of the Indonesian Seaway. Nodes C and D (Indo-Pacific vs. Atlantic Ocean): Closure of the Tethyan Seaway. Node E, F and G (amphi-Atlantic distribution patterns): continental break-up and spreading of the Atlantic Ocean. Three possible calibration ranges for the closure of the Central American Seaway were specified. Comparisons of the species affected by this vicariance event with other teleosts having similar distribution patterns (Fig. 3) indicate that the seahorses were among the last to diverge. This suggests that their cladogenesis was associated with the final closure of the seaway, i.e. no earlier than approximately 4.6 mya (a hypothesis that is further supported by the finding that marine organisms in nearshore habitats were among the last species to have diverged as a result of Central American seaway closure [20,60,61]). Divergence time estimates are indicated as mean ± S.D. (95% confidence interval). Suggested dates of vicariance events: Central American Seaway closure: 3.1 – 3.5 mya (assuming that the divergence of the transisthmian seahorse lineages took place when a land bridge formed in Central America [18]); 3.1 – 4.6 (taking into consideration that seahorse divergence may have been affected by the reorganisation of ocean currents associated with the closure of the seaway [24]); 3.1 – 8.5 mya (the upper bound being the time when the earliest recorded evolution associated with the closure of the seaway took place in marine corals and foraminiferans [25]); Indonesian Seaway closure: 0.01 – 1.8 [47,48]; 3 – 4 mya [46]; 7 – 10 mya [44,45]; 15 – 17 mya [43]; Tethyan Seaway closure: 11.2 – 14.8 mya [34,35]; 18.4 – 20.5 [36]; 23.8 – 28.5 [31]; complete separation of the land masses on either side of the Atlantic Ocean: 84 mya [50].

The establishment of the amphi-Atlantic distribution patterns all post-dated 84 mya, irrespective of the calibration range used for the closure of the Central American seaway. This suggests that these were not the result of continental break-up during the Mesozoic. Because of this, and because 95% confidence intervals of the divergence dates of nodes that have resulted in the same distribution patterns did not overlap when a calibration range of 3.1 – 3.5 mya was specified for the closure of the Central American Seaway (although some overlap was found when wider calibration ranges were specified, Table 2), interpretations of the seahorses' present-day distribution patterns based exclusively on vicariance biogeographic hypotheses are not well supported.

**Figure 4:**
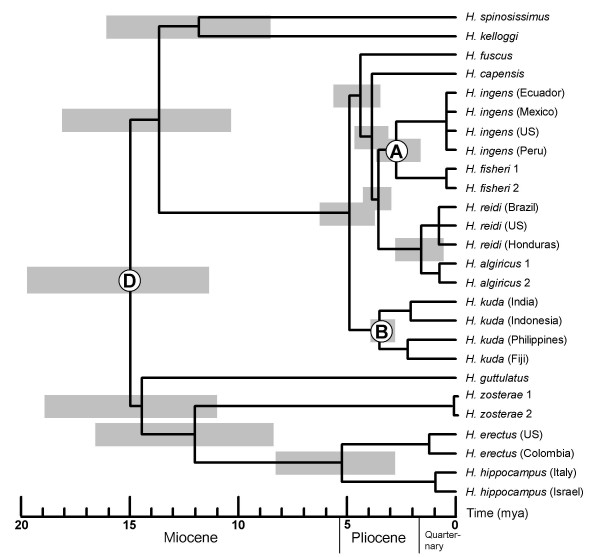
**Chronogram of the circumglobal seahorse clade**. An ultrametric tree of the circumglobally distributed seahorse lineage scaled to geological time constructed using the program MULTIDIVTIME [57]. White circles indicate nodes that were used to calibrate the molecular clock; letters within these correspond to the ones used in Fig. 2. Time intervals used for calibration were: A: 3.1 – 4.6 mya; B: 3.0 – 4.0 mya; D: 11.2 – 20.5 mya. Grey bars indicate 95% confidence intervals of internal nodes.

As divergence time estimates are more precise when multiple calibration points are specified [59], we chose a combination of vicariance events that are likely to have impacted on cladogenesis in the seahorse phylogeny to date all other nodes in the phylogeny. The selection of these was based on the assumption that the divergence event that resulted from the closure of the Central American Seaway occurred no earlier than 4.6 mya, and that the closure of the Indonesian Seaway 3 – 4 mya resulted in the divergence of the lineages defined by node B. We also included node D as a calibration point, assuming that the Atlantic Ocean vs. basally Indo-Pacific distribution of its species resulted from the closure of the Tethyan Seaway. Although the timing of this divergence event is comparatively vague, mean divergence times ± S.D. estimated when only node A (3.1 – 3.5 mya) and only node B (3.0 – 4.0 mya) were used as calibration points fell within the ranges of the two most reliable dates for this event suggested in the literature (Node A: 15.12 mya ± 3.46 mya; Node B: 14.11 mya ± 3.27 mya; Rögl and Steininger [34,35]: 11.2 – 14.8 mya; Adams et al. [32,33]: 18.4 – 20.5 mya). A phylogenetic tree scaled to geological time was constructed by specifying divergence times for the three seaway closure events (Pliocene for the Central American and Indonesian seaways and Late Early to Middle Miocene for the Tethyan Seaway) is characterized by the largest number of divergence events having taken place during the Pliocene (Fig. 4). The 95% confidence intervals of older divergence events are large compared to most of the more recent events, as we allowed for a wide calibration range for the root node.

## Discussion

### Seaway closure events as calibration points

In studies of marine organisms that employ molecular dating, it is common practice to apply the most recent dates suggested in the geological literature to date divergence events considered to have resulted from the closure of a seaway [30,60,61]. As the evolutionary histories of geminate species pairs may be more complicated than is generally acknowledged, we avoided overconfidence in calibration points by firstly determining which vicariance events were most likely to have resulted in present-day biogeographic patterns of seahorse species associated with the circumglobal clade, and secondly, by specifying upper and lower bounds for each calibration point to account for uncertainties concerning the exact dates of vicariance events. Comparisons with other teleosts showed that the amphi-Panamaic distribution pattern arose comparatively recently, and is thus likely to have been linked to the final closure of the Central American Seaway. As the four seahorse species affected by the closure of the seaway all occur in shallow water [62], this conclusion is supported by the hypothesis that divergence events among species occurring in the nearshore habitat or in freshwater are linked to the final closure of the Central American Seaway, whereas species with significant marine phases are likely to have diverged earlier [20,63,64]. Estimates of the vicariance event that resulted in the basally Indo-Pacific vs. Atlantic Ocean distribution pattern defined by node D (Figs. 2 and 4) were less certain than those of the other two vicariance events. However, our estimates indicate that this basal cladogenic event in the phylogeny is likely to have occurred some time during the Late Early to Middle Miocene, which is supported by geological estimates of the timing of the closure of the Tethyan Seaway [33-36].

Comparisons with other pairs of geminate teleost species present on either side of the Isthmus of Panama indicate that K2P distances ~0.05 for cytochrome *b*, ~0.045 for control region (domain II), and ~0.02 for 16S rRNA may indicate that the lineages under investigation are likely to have diverged as a result of the final closure of the Central American Seaway. Assuming a conservative estimate of 3.1 – 4.6 mya for the divergence of these lineages, these values correspond to approximately 1 – 1.5% (Cytochrome *b*), 0.9 – 1.4% (control region domain II) and 0.3 – 0.5% (16S rRNA) sequence divergence per million years, and are thus lower than the commonly used value of 2% per million years for animal mtDNA [65]. Our results indicate that the closure of the Indonesian Seaway may also be useful for calibrating molecular clocks. In this case, a K2P distance of ~0.03 for cytochrome *b*, ~0.036 for control region, and a distance below 0.02 for 16S rRNA may indicate that the lineages under investigation diverged as a result of Pliocene closure of the seaway.

### Causes of other dichotomies in the phylogeny

Apart from the three seaway closures, there are no obvious vicariance events that could have resulted in any of the other dichotomies. None of the divergence events resulting in sister lineages with amphi-Atlantic distributions could be linked to continental break-up (84 mya) and spreading of the Atlantic Ocean on the basis of molecular dating, even when the unlikely upper bound of 8.5 mya was specified for the closure of the Central American Seaway. This date represents the time when the constriction of the seaway and the associated increase in carbonate-content in southern Caribbean deep-sea sediments resulted in originations in reef corals and carbonate-associated benthic foraminifera, which is unlikely to have affected shallow-water seahorses. A vicariance biogeographic interpretation of these distribution patterns is further weakened by fossil data: the oldest known fossil of the family Syngnathidae (of which seahorses are considered to be one of the most derived genera by virtue of their advanced brood pouch morphology [66]) is less than 50 my old [67].

We suggest that the presence of recently diverged sister-species on both sides of the Atlantic Ocean (*H. erectus *[east coast of the Americas] and *H. hippocampus *[Europe]; *H. reidi *[east coast of the Americas] and *H. algiricus *[West Africa]), is the result of founder dispersal. Divergence time estimates for these two lineages fall into the Late Miocene/Early Pliocene and Late Pliocene/Pleistocene, respectively. Teske et al. [53] hypothesized that the ancestor of *H. hippocampus *colonized Europe from the Americas, as both its sister species (*H. erectus*) and the next basal species (*H. zosterae*) occur in the Caribbean. A Late Miocene/Early Pliocene divergence estimate confirms that this colonization may have been facilitated by an intensification of the Gulf Stream that culminated 3.8 mya [68,69]. As this change in ocean circulation was the result of the gradual closing of the Central American Seaway, the founder event that gave rise to *H. hippocampus *is thus likely to be the indirect result of tectonic changes.

The other East Atlantic species, *H. algiricus*, is also likely to be the product of long-distance dispersal in an eastward direction, as its sister species, *H. reidi*, occurs on the east coast of the Americas, and the next basal lineage comprises eastern Pacific seahorses. The divergence event that gave rise to this amphi-Atlantic geminate species pair took place more recently (Late Pliocene to Pleistocene). There are numerous examples of such recently established amphi-Atlantic distributions in the literature, and in many cases, the lineages in question are morphologically and genetically so similar that they are considered to be single species [70-73]. Even the oldest amphi-Atlantic divergence event in the phylogeny, that between the European species *H. guttulatus *and the lineage comprising basal American species (*H. zosterae *and *H. erectus*), is unlikely to be the result of vicariance following the expansion of the Atlantic Ocean, as divergence of this lineages was estimated to have occurred during the Miocene. Lastly, vicariance models invoked for the presence of species on Hawai'i suggest that because of the geological history of the Pacific plate, endemic Hawai'ian taxa should have sister taxon relationships with taxa in the Indo-West Pacific [74,75]. However, many teleost species present in the Central Pacific have sister taxon relationships with East Pacific species, and there is evidence for sporadic dispersal events in either direction [76]. Our finding that the Hawai'ian seahorse *H. fisheri *is closely associated with American/West African seahorses provides a further example of westward dispersal from the Americas, an event that was estimated to taken place after the closure of the Central American Seaway.

## Conclusion

Since the validation of plate-tectonics theory and the development of cladistic methods, vicariance events have been primarily invoked to explain disjunct species distributions throughout the world. However, improvements in molecular dating techniques have resulted in increased support for recent dispersal hypotheses over more ancient vicariance events, as in many cases, molecular divergences were considered too small to be explained by vicariance [5]. Despite these developments, Heads [12,13] rejected dispersal hypotheses in favor of vicariance hypotheses, and considered studies on the phylogeographic history of cichlid fishes [77,78] (a teleost family of Gondwanan origin that occurs exclusively in freshwater) to be exemplary in terms of their convincing conclusions (and lack of molecular dating). Given that long-distance dispersal between continents is impossible for obligate freshwater fishes, vicariance hypotheses are appropriate to explain observed distribution patterns of freshwater organisms. However, this is completely different in marine organisms, many of which can readily disperse in the oceans over great distances [27,70,71,76,79], and to whom the barrier is thus not absolute. Heads [12] acknowledged that many marine organisms can readily reach far outside their established ranges, but argued that they do not establish themselves because of competition from congeneric vicariants already established in such habitats. Likewise, Briggs [80] suggested that competition may prevent the colonization of high diversity habitats. The "competitive exclusion principle" [81] is now considered to be controversial [82]. Although a number of recent experiments do support the notion that high diversity may increase a community's invasion resistance [83-86], its effect may be difficult to discern from other factors such as predation, inappropriate climate and disease [87]. Nonetheless, the successful establishment of a founder population may be inhibited to some degree by the presence of interspecific competition [88-90], or facilitated by a lack of resource competition (i.e. the existence of an "empty niche" [91]), as the available resources allow the founders to rapidly increase their numbers. This may explain why a) the lineage defined by node A (Fig. 2) is absent from the species-rich West Pacific, despite the high dispersal potential of its species and b) the colonization of West Africa and Hawai'i (two regions with low seahorse diversity) was possible for *H. algiricus *and *H. fisheri*, respectively. The European species *H. guttulatus *and the more recent arrival *H. hippocampus *are sympatric, but they do not compete directly because of different microhabitat preferences [92]. The establishment of human-introduced marine species in new habitats is well documented [93-95], and it seems unreasonable to reject the notion that such colonization events may occur naturally, albeit at a lower rate. Both vicariance and founder dispersal thus have to be considered plausible in marine organisms, and our study indicates that molecular dating is a useful tool to determine when a divergence event is likely to have occurred and what may have caused it.

Even if one does not consider genetic differentiation of molecular markers to be at least roughly correlated with time, one must nonetheless concede that the establishment of the same phylogeographic patterns in clades nested within each other in the same phylogeny cannot have occurred simultaneously. Hence, if the divergence of *H. guttulatus *vs. *H. zosterae*, *H. erectus *and *H. hippocampus *was the result of continental break-up and spreading of the Atlantic Ocean (vicariance), then the split between *H. erectus *and *H. hippocampus *must have been caused by subsequent long-distance dispersal. This is further supported by the fact that even if the unlikely upper limit of 8.5 mya is specified for the closure of the Central American Seaway, the upper 95% confidence limit for the divergence of *H. erectus *and *H. hippocampus *postdates even the most recent estimate for the spreading of the Atlantic Ocean. Likewise, if the Atlantic biome clade comprising the above species and the basally Indo-Pacific clade comprising all other species diverged as a result of Tethyan Seaway closure, then node A cannot define a western Tethyan lineage that arose at the same time (again, the upper 95% confidence limit using the upper bound of 8.5 mya considerably postdates the most recent geological estimate for this event). Furthermore, the fact that the basal split in the seahorse phylogeny into an Atlantic Ocean lineage and a basally Indo-Pacific lineage predated all of the divergence events resulting in amphi-Atlantic distribution patterns (which could be interpreted as Tethyan Seaway closure 11.2 – 28.5 mya, and continental break-up 84 mya, respectively) suggests that none of the amphi-Atlantic distribution patterns are the result of vicariance. As none of the pairwise genetic distances between amphi-Atlantic sister lineages of other teleosts investigated were substantially greater than the distance between the two most divergent pair of seahorse lineages, we conclude that these did not diverge as a result of vicariance either. Our results thus support other recent studies on Atlantic Ocean marine organisms that identified long-distance dispersal as the cause for the establishment of amphi-Atlantic sister lineages [70,96-98].

The impression that vicariance hypotheses are increasingly being invoked to explain biogeographic patterns in the sea [12] may to some extent have been created by the increased use of molecular dating in genetic studies of marine organisms, which relies on well-documented vicariance events to use as calibration points. Founder dispersal events are less useful for this purpose, but we conclude that in the circumglobally distributed seahorse lineage, divergence events that resulted from founder dispersal are likely to outnumber divergence events that resulted from vicariance. We hypothesise that founder dispersal is thus of particular importance in species that disperse by means of rafting.

## Methods

### Taxon sampling and sequencing

The total sample used to reconstruct the phylogeny of the monophyletic circumglobally distributed seahorse lineage (clade 4 in Teske et al. [53]) consisted of 26 individuals from 13 species (Table 3). We also included a single individual each of species from the circumglobal clade's three sister lineages (clades 1, 2 and 3 in Teske et al. [53]) as outgroup taxa. We attempted to obtain tissue material from more than one specimen of each ingroup species to account for intra-specific variation. This was considered particularly important in the case of species with wide distribution ranges. The Hawai'ian seahorse that was previously referred to as *Hippocampus hilonis *[6] or *H. fisheri *[53] is correctly referred to as *H. fisheri *in this study. Morphologically, this species resembles *H. trimaculatus *(a species closely related to the outgroup species *H. comes*), suggesting that it is not part of the circumglobal clade and that specimens that group genetically with *H. ingens *are likely to be *H. hilonis *(a possible synonym of *H. kuda*, SA Lourie, pers. comm.). We have now confirmed that our Hawai'ian specimens are morphologically very different from *H. kuda *and that they fit the descripton of *H. fisheri *[62] well. All five genetic markers used in this study confirm that our specimens are the sister taxon of *H. ingens*, that they are not part of the *H. kuda *complex, and that they are genetically very different from *H. trimaculatus *(Fig. 2). This suggests that the morphology of *H. fisheri *was misleading.

For phylogenetic reconstruction and molecular dating, we used sequence data of three mitochondrial markers (control region, cytochrome *b *gene and 16S rRNA) and one nuclear marker (the first intron of the S7 ribosomal protein, herafter referred to as S7 intron). Sequences of a second nuclear marker, Aldolase, were used for phylogenetic reconstructions only. Mitochondrial markers often fail to resolve deeper relationships at the taxonomic levels of family and order in various teleosts [99,100], but generally provide good resolution at and below the genus level [101,102]. For that reason, they can be considered ideal to study the phylogeny of the circumglobal seahorse clade (one of five major genetic lineages comprising the genus *Hippocampus *[53]). DNA extraction and amplification of molecular markers followed previously described protocols [43,53,103].

**Table 3:** Samples used in this study, including species names, collection localities, collectors or museums that contributed samples and GenBank accession numbers.

			GenBank accession numbers				
			
Species	Collection locality	Collector/Museum	Control region	Cytochrome b	16S rRNA	S7 intron	Aldolase
Ingroup:							
*Hippocampus*	Benin (*Ghana)	Z. Sohou,	DQ288337	AF192642*	AY277302	AY277328	AY277366
*algiricus*	Benin	*J. Macpherson	DQ288338	DQ288353	AY277302	AY277328	
*H. capensis*	South Africa	P. Teske	AY149667	AF192650	AY277304	AY277331	AY277357
*H. erectus*	USA (Gulf of Mexico)	FM	DQ288325	AF192662	AF355007	AY277339	AY277354
	Colombia	H. Hamilton	DQ288326	DQ288341	DQ288359	DQ288378	
*H. fisheri*	Hawaii	H. Hamilton	AY642331	DQ288350	DQ288369	AY277340	AY277358
	Hawaii	H. Hamilton	AY642331	DQ288351	DQ288370	AY277340	
*H. fuscus*	Egypt (Red Sea)	H. Gabr	AY642337	DQ288354	DQ288371	AY277335	AY277359
*H. guttulatus*	Italy (*Portugal)	PS, *J. Curtis	DQ288322	AF192664	AY277307	AY277337	AY277361*
*H. hippocampus*	Italy (Portugal*)	PS	DQ288323	AF192666	AY277306	AY77338	AY277374*
	Israel (Mediterranean)	B. Galil	DQ288324	DQ288340	DQ288358	DQ288377	
*H. ingens*	Ecuador	H. Hamilton	DQ288333	DQ288346	DQ288365	DQ288383	
	Mexico (East Pacific)	J. Baum	AY642329	DQ288345	DQ288364	AY277334	
	Peru	PS	DQ288331	AF192672	AY277303	AY277333	AY277365
	USA (East Pacific)	H. Hamilton	DQ288332	DQ288344	DQ288363	DQ288382	
*H. kelloggi*	Vietnam	PS	AY629249	AF192675	AY277298	AY277325	AY277350
*H. kuda*	Fiji	H. Hamilton	AY642333	DQ288357	DQ288374	DQ288388	
	India	A. Sreepada	AY642345	AF192679	DQ288372	AY277324	AY277355
	Indonesia	S. Lourie	AY642356	DQ288356	DQ288373	DQ288387	
	Philippines	M. Santos	AY642369	AF192683	DQ288375	AY277329	AY277356
*H. reidi*	Brazil (aquarium trade)	L. Smith	DQ288336	AF196292	DQ288368	DQ288386	
	Honduras	H. Hamilton	DQ288335	DQ288348	DQ288367	DQ288385	
	USA (Gulf of Mexico)	PS	DQ288334	DQ288347	DQ288366	DQ288384	AY227367
*H. spinosissimus*	Philippines	S. Lourie	DQ288329	AF192695	AY277296	AY277323	AY277364
*H. zosterae*	USA (Gulf of Mexico)	FM	DQ288327	AF356071	DQ288360	DQ288379	AY277371
	USA (Gulf of Mexico)	FM	DQ288328	AF356071	DQ288361	DQ288380	
Outgroup:							
*H. breviceps*	Australia	AM	DQ288319	AF192647	AY277287	AY277320	AY277342
*H. comes*	Philippines	N. Perante	DQ288321	AF192656	AY277289	DQ288376	AY277352
*H. coronatus*	Japan	T. Mukai	DQ288320	AF192658	AY277293	AY277319	AY277348

Sequences in boldface were used to reconstruct phylogenies. All sequences except those of Aldolase were used for molecular dating. Cytochrome *b *sequences whose accession numbers start with AF are 423 bp longer than those starting with DQ and were used to reconstruct phylogenies. Control region, cytochrome *b *and 16S rRNA are mitochondrial markers, S7 intron and Aldolase are nuclear markers. In some cases, samples from two different localities were used to represent a particular species.

FM = Florida Museum, PS = Project Seahorse, AM = Australian Museum

*Collection locality, collector, and accession number of the less frequently used sample

A total of 61 new sequences were generated for this study (GenBank accession numbers starting with DQ, Table 3). These were complemented with 82 previously published seahorse sequences (accession numbers starting with AF and AY [6,43,52,53]). For phylogenetic reconstructions, complete cytochrome *b *sequences generated by Casey et al. [52] were used whenever available, which are 424 bp longer than the partial cytochrome *b *sequences generated in this study and in Lourie et al. [43]. Aldolase sequences were obtained for a single individual of each species only. As this marker was characterized by comparatively little variation, it was not used for molecular dating.

### Alignments and phylogenetic reconstructions

Sequence alignments were generated using BALI-PHY [104]. This program estimates alignment and phylogeny simultaneously in a Bayesian framework, and in this way avoids the problems associated with poor guide trees in the more widely used alignment program CLUSTALX [105]. Confidence of the results is assessed using posterior probabilites, and the indel model implemented allows indels several characters in length and also allows these to nest or overlap if they lie on separate branches. Three partitions were characterized by length differences (control region, 16S rRNA and S7 intron). These were aligned individually because of computational constraints when aligning combined data-sets. Prior to generating BAli-PHY alignments, we explored which models of sequence evolution were most appropriate for each partition by generating CLUSTALX [105] alignments of ingroup sequences using default settings, and then using the Akaike information criterion [106] as implemented in MODELTEST version 3.7 [107]. As all models selected were fairly complex, we specified the most complex model presently implemented in BALI-PHY (the Tamura-Nei model [108]) for simultaneous estimation of alignment and phylogeny. A gamma distribution parameter and an assumed proportion of invariable sites were also specified if these were selected as model components by MODELTEST. For each partition aligned in BALI-PHY, 500 iterations were performed, and the final alignment was based on all alignments recovered excluding the burn-in. The procedure was repeated five times to ensure consistency of results. BALI-PHY's algorithm has very short burn-in times, and in the case of our comparatively small data-sets, convergence was complete by the tenth generation for all three partitions characterized by length differences. Maximum *a posteriori *alignments used for further analyses were thus based on the remaining 490 iterations.

A single representative of each species was used for alignment and phylogenetic reconstruction, because exploratory alignments using the program CLUSTALX [105] followed by phylogenetic reconstructions using the neighbour-joining method [109] revealed that the geographically distant representatives of each species were monophyletic. An exception was made in the case of the Indo-Pacific species *Hippocampus kuda*, which was represented by one individual from India (Indian Ocean lineage) and one individual from north Sulawesi, Indonesia (West Pacific lineage). Control region sequences indicated that these two lineages may not be monophyletic and might be considered to be different species [6], a result that was, however, not strongly supported. In addition to 14 specimens representing the ingroup, we also included the three outgroup species *H. breviceps*, *H. comes *and *H. coronatus *(representatives of clades 1, 2 and 3 in Teske et al. [53]). As the alignment method was strongly influenced by the presence of missing data, a section of missing data 50 bp in length in the S7 intron sequence of *H. erectus *was temporarily replaced with corresponding characters from its sister species *H. hippocampus *(the surrogate characters were removed for subsequent analyses). The sister-taxon relationship of the two species was strongly supported by previous studies [52,53].

The data matrix used for phylogenetic reconstructions included the three partitions aligned using BALI-PHY, as well as three additional partitions. Firstly, complete cytochrome *b *sequences were used, whenever available (resulting in 424 bp of missing data in *H. fisheri *and *H. fuscus*). Secondly, indels in the three partitions that were characterized by length differences were coded as presence/absence characters in some analyses if they were present in more than one species, had a length of more than one nucleotide, and had clearly defined alignment boundaries. Thirdly, Aldolase sequences [53] were included (Table 3). These contained only two indels, each of them one nucleotide in length, and were aligned by eye. The aligned sequence lengths of control region, 16S rRNA and S7 intron were 389, 532 and 596 nucleotides in length, respectively. Eight indels were coded as presence/absence data, and cytochrome *b *and Aldolase sequences were 1140 and 188 nucleotides in length, respectively, resulting in a total sequence length of 2853 characters.

This data matrix (excluding the partition comprising indels) was used to recover a maximum likelihood tree by generating 200 iterations of the 'likelihood ratchet' [110], which is a model-based procedure analogous to the parsimony ratchet [111]. Tree searches that employ ratcheting are less likely to become stuck on suboptimal tree islands than any other method of phylogenetic reconstruction presently available. Runs were repeated five times to ensure that the tree space was adequately explored, as indicated by the tree topology with the highest likelihood score being consistently recovered. Nodal support for this tree was obtained by means of bootstrap resampling (1000 replicates) using maximum likelihood in PAUP* version 4.0b10 [112], with a single most appropriate model being specified for the whole data-set as determined using the Akaike Information Criterion in MODELTEST. The heuristic search was limited to a maximum of 10 000 saved trees. Secondly, we used the heuristic parsimony analysis in PAUP* to recover the most parsimonious tree using the same data-set and including indels as presence/absence characters. Default parameters were specified, with 100 random addition replicates and 1000 trees retained at each step. Nodal support for this topology was obtained by means of jackknifing (100 000 replicates, 50% deletion) using default parameters in PAUP*. Thirdly, MRBAYES version 3.1 [113] was used to determine posterior probabilities for each node. The Markov chain Monte Carlo process was set for four chains to run simultaneously for 2 000 000 generations, with trees being sampled every 100 generations. In addition to examining posterior probabilities of the resulting trees to determine when burn-in was complete, we also compared standard errors of posterior probabilities between simultaneous runs. As these tended to decrease for some time after the burn-in phase, only trees were used once the difference in standard errors had also stabilized (i.e. the first 4 000 out of a total of 20 000 trees were exluded). Bayesian analyses were repeated three times to ensure that chains had converged. We specified unique model priors for each partition as determined using MRMODELTEST version 2.2 [114]. An exception were the 16S rRNA sequences, which were aligned to the secondary structure model of the teleost *Pygocentrus nattereri *[115] to identify stem and loop regions, and the doublet model was invoked for complementary stem regions. Model priors from MRMODELTEST were, however, specified for stem regions for which no complementary regions were available for our partial 16S rRNA sequences, as well as for loop regions.

### Molecular dating

We compared genetic divergence between seahorse lineages whose distribution patterns may have arisen as a result of the closures of the Central American, Tethyan or Indonesian seaways, or spreading of the Atlantic Ocean, with those of various other teleost lineages having the same distribution patterns. Following previous studies that estimated genetic distances of transisthmian sister species [22,23], we calculated genetic distances under the Kimura 2-Parameter (K2P) model [116]. As cytochrome *b *and 16S rRNA sequences are most frequently used for phylogenetic reconstructions in teleosts, we limited our comparisons to these two markers.

To ensure that evolutionary rates between seahorses and other teleosts were not significantly different, we applied the relative rate test implemented in RRTREE [117]. This test takes into account phylogenetic relationships and corrects for sampling imbalances. All relative rate tests were carried out in a pairwise fashion by comparing rates in the seahorse phylogeny with rates in one of the other teleost lineages. As outgroup for each pairwise comparison, we used the bonefish *Albula*, as this was the only non-percomorph teleost genus investigated. In the case of cytochrome *b*, tests were performed separately for synonymous and non-synonymous substitutions by computing the parameters B4 (number of synonymous transversions per fourfold degenerate site) and Ka (number of non-synonymous substitutions per non-synonymous site), whereas the K2P substitution model was applied to the 16S rRNA data (using BALI-PHY alignments of seahorses and other teleosts that were generated as described previously). For comparison, we also plotted pairwise distances of lineages of seahorses and other teleosts that may have arisen as a result of the closures of the Indonesian and Tethyan seaways, as well as continental break-up followed by spreading of the Atlantic Ocean, by virtue of their present-day distribution patterns. Lineages characterized by different evolutionary rates than the seahorses, as well as lineages that were not tested either because different positions of a particular molecular marker were used or because they were used as outgroup for the relative rate test were also plotted for comparison, but were not considered in the analyses.

To date nodes in the seahorse phylogeny, we explored whether rate differences were present among species using RRTREE. The same three species that comprised the outgroup in phylogenetic reconstructions were also used as reference taxa in these analyses. Significant rate differences were identified in the case of three of the partitions (16S rRNA, control region and S7 intron), which prompted us to estimate the ages of divergence events among different seahorse lineages by means of the Bayesian relaxed clock method for multiple genes implemented in the programmes ESTBRANCHES and MULTIDIVTIME [58]. The maximum likelihood tree topology was specified, and to incorporate within-species differences, we included all specimens available for each species and specified phylogenetic relationships among these as unresolved polytomies. Again, the three species *Hippocampus breviceps*, *H. coronatus *and *H. comes *were used as outgroup taxa. MULTIDIVTIME was used to estimate the ages of divergence events and their 95% confidence intervals. The Markov chain was sampled 50 000 times, with 100 cycles between each sample and a burn-in of 50 000 cycles. The process was then repeated by sampling 100 000 times and specifying a burn-in of 100 000 cycles. Differences in the results of these two runs would indicate that the program has not been run for sufficiently long for Markov chains to converge. This was found not to be the case (divergence time estimates differed by no more than 0.0005).

Settings of the parameters *rttm*, *rtrate*, *brownian *and *big time *followed suggestions in the MULTIDIVTIME manual. The prior expected number of time units between tip and root (*rttm*) was set to 22 mya (Early Miocene), as this date was between the most recent (Middle Miocene) and oldest (Late Oligocene) divergence times specified for the root node. As the value for *rttm *should be between 0.1 and 10, we set it to 2.2. All subsequent values expressing time in million years were also multiplied by 0.1.

The mean of the prior distribution for the rate at the root node (*rtrate*) was estimated by dividing the median of all branch lengths from root to ingroup tips by *rrtm*. A value of 0.7 was specified for *brownian*, as *brownian *multiplied by *rttm *should be between 1 and 2 (2.2 × 0.7 = 1.5). The highest possible number of time units between tip and root (*big time*) was set to 50 (i.e. 500 million years). The magnitude of standard errors was set equal to that of each of the parameters *rttm*, *rtrate *and *brownian*. We examined the effect of the choice of parameter priors on divergence time estimates by doubling all of them. The fact that time estimates obtained in this way differed by no more than 0.1 (1 million years; mean difference: 0.03 or 300 000 years) suggests that the choice of priors had little effect on time estimates.

Molecular dating was done in two ways. Firstly, to date divergence events that may have resulted from vicariance events, we specified only the well-documented closure of the Central American Seaway as a calibration point and specified three alternative calibration ranges for the timing of this event: a) 3.1 – 3.5 mya [19], i.e. assuming that the divergence of the transisthmian seahorse lineages took place during the final closure of the seaway; b) 3.1 – 4.6 mya, with the upper limit representing the onset of a marked reorganization of ocean circulation in Central America that was associated with the rising of the Isthmus of Panama [35], and c) 3.1 – 8.5 mya, to account for the possibility that seahorses were affected by the earliest possible evolution associated with the closure of the seaway [26]. Secondly, we dated all nodes in the phylogeny by using a well-supported divergence range of the transisthmian seahorse lineages in combination with other divergence events whose divergence time estimates matched the timing of vicariance events suggested in the literature in the first analysis.

## Authors' contributions

PRT designed the study, obtained the majority of the samples, carried out the laboratory work, did the analyses and prepared the manuscript. HH provided additional samples and helped with the laboratory work. CAM and NPB participated in the design of the study and contributed to the preparation of the manuscript. All authors read and approved the final version.
